# Genetic variants and physical activity interact to affect bone density in Hispanic children

**DOI:** 10.1186/s12887-021-02537-y

**Published:** 2021-02-15

**Authors:** Ruixue Hou, Shelley A. Cole, Mariaelisa Graff, Yujie Wang, Karin Haack, Sandra Laston, Nitesh R. Mehta, Roman J. Shypailo, Margaret L. Gourlay, Anthony G. Comuzzie, Kari E. North, Nancy F. Butte, Venkata Saroja Voruganti

**Affiliations:** 1 Department of Nutrition and Nutrition Research Institute, University of North Carolina at Chapel Hill, 500 Laureate Way, Kannapolis, NC 28081 USA; 2 Texas Biomedical Research Institute, San Antonio, TX USA; 3 Department of Epidemiology, University of North Carolina at Chapel Hill, Chapel Hill, NC USA; 4 South Texas Diabetes and Obesity Institute and Department of Human Genetics, University of Texas of the Rio Grande Valley, Brownsville, TX USA; 5 USDA/ARS Children’s Nutrition Research Center, Department of Pediatrics, Baylor College of Medicine, Houston, TX USA; 6 Department of Family Medicine, University of North Carolina at Chapel Hill, Chapel Hill, NC USA; 7 The Obesity Society, Silver Spring, MD USA

## Abstract

**Background:**

Our aim was to investigate if moderate to vigorous physical activity (MVPA), calcium intake interacts with bone mineral density (BMD)-related single nucleotide polymorphisms (SNPs) to influence BMD in 750 Hispanic children (4-19y) of the cross-sectional Viva La Familia Study.

**Methods:**

Physical activity and dietary intake were measured by accelerometers and multiple-pass 24 h dietary recalls, respectively. Total body and lumbar spine BMD were measured by dual energy X-ray absorptiometry. A polygenic risk score (PRS) was computed based on SNPs identified in published literature. Regression analysis was conducted with PRSs, MVPA and calcium intake with total body and lumbar spine BMD.

**Results:**

We found evidence of statistically significant interaction effects between the PRS and MVPA on total body BMD and lumbar spine BMD (*p* < 0.05). Higher PRS was associated with a lower total body BMD (β = − 0.040 ± 0.009, *p* = 1.1 × 10^− 5^) and lumbar spine BMD (β = − 0.042 ± 0.013, *p* = 0.0016) in low MVPA group, as compared to high MVPA group (β = − 0.015 ± 0.006, *p* = 0.02; β = 0.008 ± 0.01, *p* = 0.4, respectively).

**Discussion:**

The study indicated that calcium intake does not modify the relationship between genetic variants and BMD, while it implied physical activity interacts with genetic variants to affect BMD in Hispanic children. Due to limited sample size of our study, future research on gene by environment interaction on bone health and functional studies to provide biological insights are needed.

**Conclusions:**

Bone health in Hispanic children with high genetic risk for low BMD is benefitted more by MVPA than children with low genetic risk. Our results may be useful to predict disease risk and tailor dietary and physical activity advice delivery to people, especially children.

**Supplementary Information:**

The online version contains supplementary material available at 10.1186/s12887-021-02537-y.

## Introduction

Osteoporosis and low bone mass are major public health threats with great economic burden in the U.S. Maximizing peak bone mass in early life is an important strategy to prevent osteoporosis in later life. Bonjour et al. (2009) reported that an increase of peak bone mass in childhood by one standard deviation could reduce fracture risk as much as 50% in adulthood [1]. Physical activity and calcium intake are two of the most important modifiable factors for bone health and their benefits to bone health could persist into young adulthood [2, 3]. Ethnic-wise distribution shows that Hispanic adolescents are less physically active, and have lower calcium intake compared to their White counterparts [4, 5]. Despite their importance, the role of physical activity and calcium intake with respect to genetic susceptibility to low bone mass has not been sufficiently studied in Hispanic children.

Studies that account for the full complexity of genetic and environmental factors influencing BMD have been mostly conducted in adults typically focusing on few candidate genes, with very few studies being conducted in children or capturing the effects of multiple genes [6, 7]. Polygenic risk scores (PRSs), which could capture the genetic effects of multiple genes, could be a better approach to assess risk for osteoporosis. In one study of European children, significant interaction was found between physical activity and a PRS computed from selected SNPs identified from published genome-wide association studies (GWAS) [8]. However, none of these studies have been conducted in a Hispanic population nor investigated genome-wide SNPs to identify significant genotypes that would interact with physical activity or calcium intake to affect BMD. Therefore, our aim was to examine if physical activity or calcium intake interact with BMD-associated PRS to affect BMD in an understudied and underserved population of Hispanic children aged 4–19 years old.

## Methods

### Study population: the viva La Familia study (VFS)

The VFS, a family study of 1030 Hispanic children (4-19y) from 319 families enrolled in Houston, TX, was designed to examine the genetic and environmental factors influencing obesity and its comorbidities, with the majority of families of Mexican-American descent [9, 10]. Each family was chosen from an overweight proband (4-19y) with the use of a bivariate procedure, ie, overweight ≥95th percentile for BMI and ≥ 85th percentile for fat mass. In addition, it was required to have ≥3 children between the ages of 4 and 19 y within the families [9, 10]. Related families were also recruited to expand the relative pair types. Interviews were conducted to obtain family pedigree, socio-demographics, lifestyle information, and medical histories at baseline. Puberty stage was self-reported in children. Anthropometrics were obtained from children and their parents [9]. Dual energy X-ray absorptiometry (DXA) with a Delphi-A whole-body scanner (Hologic Inc., Waltham, MA) was used to obtain total body and lumbar spine BMD. Data analysis was conducted in about 750 children for whom complete baseline data (genetic data, dietary intake, physical activity and bone measurements) were available.

Written informed consent or assent was given by all enrolled children’ s parents. The protocol was approved by the Institutional Review Boards for Human Subject Research at Baylor College of Medicine and Affiliated Hospitals and Texas Biomedical Research Institute and the analysis was approved by the University of North Carolina at Chapel Hill. All methods were performed in accordance with the relevant guidelines and regulations.

### Physical activity

Actiwatch accelerometers (Mini Mitter, Bend, OR) were used to monitor free-living physical activity continuously for 3 days, with details previously described [11], yielding activity counts at 1 minute intervals. Using thresholds corresponding to physical activity ratios of 1.5, 3.0, and 6.0 or energy expenditure of 0.01, 0.04, and 0.10 kcal/kg per minute, based on validated study in children [12]. Moderate to vigorous activity (MVPA) is more likely to capture the weight-bearing physical activity than light or sedentary physical activity, therefore we chose MVPA (mins/day) as a key measure for physical activity in this study.

### Dietary intake

A multiple-pass 24 h dietary recall was recorded on 2 occasions, ~ 2–4 weeks apart, at the Children’s Nutrition Research Center by a registered dietitian using Nutrition Data System Software (database version, 2005, Nutrition Coordinating Center, University of Minnesota, Minneapolis) and food models and household measures/dishware. This system automates interviewing, editing, and coding of dietary intake data. The multiple-pass 24 h dietary recall method uses 3 distinct passes to garner information about a subject’s food intake during the preceding 24 h. The 24-h recalls were obtained without prior notice and children aged < 7 y were assisted by their mothers. Mean nutrient intakes were used in the analysis, computed from the two 24-h dietary recalls [13].

### Genotyping of common variants and sequencing of exome variants

For common variants, genotyping for 1.1 million SNPs was conducted using marker assays included on the Illumina HumanOmni1-Quad v1.0 BeadChips, with details described previously [14]. For exome sequencing, the custom NimbleGen VCRome 2.1 capture reagent was used to capture the entire exome for each DNA sample and details have been previously published [14].

### Covariates

Weight was measured with a digital balance to the nearest 0.1 kg, and height was measured to the nearest 1 mm with a stadiometer. BMI was calculated as kg/m^2^ and BMI z-score was calculated based on Centers for Disease Control and Prevention (CDC) [15] standardized sex and age growth charts on the basis of the L (normality) M (median) S (dispersion) formula:

$$ z\  score=\left(\frac{BMI^L}{M}\right)\div \left(L\ x\ S\right) $$

Tanner stages of maturation were self-reported based on pubic hair and breast and penis development illustrated with drawings [9]. Puberty stages were defined as pre-pubertal (Tanner stage = 1) and pubertal (Tanner stage > 1) [16].

### Fine mapping

Index SNPs were independent SNPs that were associated with total body BMD and lumbar spine BMD in a recent paper focusing on life-course genome-wide meta-analysis of bone health [17, 18]. Within a ± 500 kb region of each index SNP, SNPs with the lowest *p* value in VFS (“VFS_SNP”) were identified. Linkage disequilibrium (LD) between the index SNPs and the corresponding “VFS_SNP” were computed by LD link (https://ldlink.nci.nih.gov) in corresponding ethnic populations in which the index SNPs were originally reported. LD (r^2^) greater than 0.2 was considered as the threshold that SNPs in VFS might be a better signal of index SNPs. Proxy SNPs (r^2^ > 0.8 based on LD link, reference population: CEU) within the ±500 kb region of the index SNPs were also identified, when index SNPs found in literature were not available in VFS data. Within each 1 Mb region, the number of independent SNPs was calculated based on LD structure between SNPs in our cohort. The *p* value threshold for each 1 Mb region was determined using the Bonferroni adjustment (*p* value threshold).

### Polygenic risk score (PRS) computation

SNPs of PRS include: 1) SNPs that are statistically significant or nominally significant per GWAS threshold (*p* < 1.0 × 10^− 7^ and *p* < 1.0 × 10^− 6^, respectively) and exome sequencing analysis (*p* < 9.2 × 10^− 7^ and *p* < 9.2 × 10^− 6^, respectively) [14]; 2) Index SNPs or Proxy SNPs in 1 Mb region that were statistically significantly and nominally significantly associated with bone health (*p* < *p* value threshold and *p* < 0.05, respectively). Nominally significantly associated-SNPs were included as these SNPs may have reached genome-wide significance if we had greater power, and more powerful PRS typically are constructed by incorporating both suggestive and significantly associated SNPs [19].; 3) “VFS_SNP” that have no strong LD (r^2^ < 0.2) with index SNPs but were statistically significantly associated with bone outcomes (*p* < *p* value threshold); 4) When “VFS_SNP” had strong LD (r^2^ > 0.2) with index SNPs and were statistically significantly associated with bone outcomes, we conducted conditional analyses including both proxy SNPs and “VFS_SNP” to determine the better signal in place of the Index SNPs, and the better one was included in PRS. As genetic variants related to BMD are site specific [20], separate PRS for total body BMD and lumbar spine BMD were computed, with details shown in Table 1**.** In addition, we conducted sensitivity analysis with PRS that included only statistically significant SNPs (secondary PRS), with details shown in Additional file 1. Minor allele frequencies of selected SNPs were above 0.01. Each SNP was recoded as 0, 1, or 2 based on the number of risk alleles (associated with lower BMD). PRS was constructed by summing up the risk alleles at each locus assuming equal effects at each locus: PRS = SNP_1_ + SNP_2_ + … + SNP_n_.

**Table 1:** Polygenic Risk Score Compositions^1, 2^

Marker	Gene	Risk Allele (Freq)	Chr	Position
**TBBMD PRS**				
rs452369	*SLC8A1*	A(0.24)	2	40,157,593
rs529125	*SUPT3H*	A(0.87)	6	44,829,351
rs2800718	*LOC105377989*	A(0.21)	6	127,079,180
rs10953178	*SEM1*	G(0.36)	7	96,501,326
rs950083		C(0.74)	10	122,717,980
rs12273330	*DGKZ*	A(0.03)	11	46,340,356
rs4394609		A(0.93)	1	22,406,025
rs914153		A(0.70)	21	38,980,535
rs370055571	*MAP 4 K3*	A(0.01)	2	39,258,582
**LBBMD PRS**				
rs9846561	*LEKR1*	A(0.96)	3	157,040,993
rs16934799	*KCNMA1*	A(0.98)	10	77,448,298
rs2087058	*LOC105369777*	G(0.54)	12	54,199,621
rs1286083	*RPS6KA5*	G(0.12)	14	90,976,435
rs7071206		A(0.75)	10	77,641,558
rs6684375		G(0.90)	1	22,379,941

^1^ Polygenic Risk Score includes SNPs that are statistically significant or nominally significant associated with BMD in Viva La Familia Study

^2^ *BMD* bone mineral density, *DGKZ* Diacylglycerol Kinase Zeta, *PRS* Polygenic Risk Score, *KCNMA1* Potassium Calcium-Activated Channel Subfamily M Alpha 1, *LEKR1* Leucine, Glutamate and Lysine Rick 1, *MAP 4K3* Mitogen-Activated Protein Kinase Kinase Kinase Kinase 3, *RPS6KA5* Ribosomal Protein S6 Kinase A5, *SEM1* 26S Proteasome Complex Subunit, *SLC8A1* Solute Carrier Family 8, *SUPT3H* SPT3 Homolog, SAGA and STAGA Complex Component

### Statistical analysis

Total body BMD and lumbar spine BMD were inverse normalized to attain normal distribution in the analysis [21, 22]. PRS was used as continuous variables as we postulated a linear relationship, but we also used binary variables categorized based on medians (> = median vs below) to test sensitivity of results. Environmental variables: dietary intake (calcium, calcium to phosphorous ratio) and MVPA (mins/d) were included alternatively as continuous variables or binary variables categorized based on median (> = median vs below) [23]. Covariates included age, sex, BMI Z-score, and puberty stage. As BMD tends to be lower for smaller bones compared to larger bones [24], we considered adding height into the current model for sensitivity analysis. However, as height was strongly correlated with age in our cohort (r = 0.91, *p* value < 0.001), we did not add height as a covariate, in addition to age, in the model to avoid issues of collinearity.

First, main effects were examined by conducting association analysis between environmental variables and BMD with linear regression. For the gene-environment interaction analysis, the modified generalized estimating equation was used and the regression model included the PRSs, the environment variables (calcium intake, calcium/phosphorus, MVPA, alternatively), the interactions term between the PRS and environment variables, and other covariates (age, sex BMI Z-score and puberty stage). For interaction analysis between PRS and environmental factors, the statistical significance level was considered as *p* < 0.05. Interactions between individual variants and environmental factors were considered as secondary analyses. Statistical analyses were performed using the software SUGEN (http://dlin.web.unc.edu/software/SUGEN/) [25].

## Results

The characteristics of VFS participants are shown in Table 2 and Additional file 1. The children in VFS have a mean age and BMI of 11.0 ± 4.1 y and 25.1 ± 7.6 kg/m^2^, respectively. The mean calcium intake was 881.5 ± 377.3 mg with a median of 841.5 mg, which is lower than recommended dietary allowances for calcium [26]. The mean dietary calcium/phosphorous ratio was 0.75 ± 0.19 with a median of 0.75, which is lower than the dietary optimal ratio of 1.3 [27]. The mean MVPA was 82.3 ± 51.8 mins/d with a median of 74 mins/d, which is higher than the recommended physical activity level for youth [28]. The lower TBBMD and LSBM observed in high MVPA group could be potentially due to younger age and higher number of pre-pubertal children in high MVPA group. The characteristics of VFS children by PRS groups are shown in Additional file 1.

**Table 2:** Baseline characteristics of children in the Viva La Familia Study^1^

	Overall	High Calcium	Low Calcium	High PA	Low PA
Age, years	11.0 ± 4.1	10.91 ± 3.82	11.11 ± 4.15	9.2 ± 3.4	*12.7 ± 3.5
BMI, kg/m^2^	25.1 ± 7.6	25.01 ± 7.50	25.26 ± 7.67	23.6 ± 7.2	*26.9 ± 7.7
Puberty Stage, Pre-pubertal %	45.3	47.3	42.4	63	*26
**Dietary Intake**					
Calcium intake, mg	881.5 ± 377.3	1176.26 ± 284.19	*586.67 ± 173.54	889.0 ± 360.5	879.9 ± 392.1
Phosphorus intake, mg	1180.3 ± 407.8	1436.63 ± 343.49	*915.30 ± 263.41	1159.4 ± 382.8	1195.0 ± 422.3
Dietary Calcium/Phosphorus ratio	0.75 ± 0.19	0.83 ± 0.15	*0.66 ± 0.17	0.77 ± 0.18	0.73 ± 0.19
**Physical Activity (PA)**					
Moderate and Vigorous, mins/d	82.3 ± 51.8	84.10 ± 51.86	80.38 ± 51.15	123.1 ± 41.3	*42.3 ± 19.5
**Bone Mineral Density**					
TBBMD, g/cm^2^	0.918 ± 0.140	0.917 ± 0.140	0.915 ± 0.139	0.868 ± 0.120	*0.974 ± 0.135
LDBMD, g/cm^2^	0.805 ± 0.184	0.795 ± 0.181	0.812 ± 0.184	0.731 ± 0.150	*0.887 ± 0.181

^1^Values are means ± SDs or percentages

*Indicated statistically significant differences between the groups (*p* < 0.05)

### Moderate to vigorous physical activity (MVPA) – genetic interaction on BMD

No statistically significant associations were found between MVPA and BMD or genetic variants and MVPA. However, we found statistically significant effects of interactions between binary MVPA (mins/d) and total body BMD PRS on total body BMD (*p* = 0.005) (Table 3). Children with low MVPA (mins/d) on average have 0.040 g/cm^2^ total body BMD less in the low PRS group as compared to the high PRS group, whereas children with high MVPA (mins/d) on average have 0.015 g/cm^2^ total body BMD less in the low PRS group as compared to the high PRS group. We also found statistically significant interactions between binary MVPA (mins/d) and total body BMD PRS in relation to lumbar spine BMD (*p* = 0.001) (Table 3). Children with low MVPA (mins/d) on average have 0.042 g/cm^2^ lumbar spine BMD less in the low PRS group as compared to the high PRS group, whereas children with high MVPA (mins/d) on average have 0.008 g/cm^2^ lumbar spine BMD less in the low PRS group as compared to the high PRS group. In further analysis with individual SNPs included in the PRS, a statistically significant interaction was found between MVPA (mins/d) and rs10953178 at 26S Proteasome Complex Subunit (*SEM1*) (*p* = 0.046) and rs914153 (*p* = 0.028) to affect total body BMD, and rs452369 at Solute Carrier Family 8 Member A1 (*SLC8A1*) (*p* = 0.038), rs10953178 (*SEM1*) (*p* = 0.0074) and rs914153 at chromosome 21q21 (*p* = 0.0058) to affect lumbar spine BMD (Table 3). In the sensitivity analysis using secondary PRS, similar results were found (Additional file 1).

**Table 3:** Interaction between PRS and moderate to vigorous physical activity on TBBMD and LSBMD ^1,2,3^

Marker	β_G^4^	*P*value_G	β_Interaction^5^	*P*value_Interaction
**Polygenic Risk Scores**				
**TBBMD**				
TBBMD_PRS, cat	−0.5	5.9 × 10^−5^	0.37	0.0047
**LSBMD**				
TBBMD_PRS, cat	−0.45	1.8 × 10^−5^	0.36	0.0011
**Individual SNPs**				
**TBBMD**				
rs10953178	−0.29	7.6 × 10^−4^	0.22	0.046
rs914153	−0.21	5.5 × 10^−3^	0.2	0.028
**LSBMD**				
rs452369	−0.38	5.6 × 10^−6^	0.24	0.038
rs10953178	−0.26	1.5 × 10^− 3^	0.27	0.0074
rs914153	−0.23	6.2 × 10^−4^	0.24	0.0058

^1^ Only results with statistically significant interactions are shown (*p* value for interaction< 0.05)

^2^ Adjusted for age, sex, BMI Z-score, and puberty stage

^3^ *PRS* Polygenic Risk Score, *LSBMD* lumbar spine bone mineral density, *TBBMD* total body bone mineral density; *TBBMD_PRS, cat* TBBMD unweighted PRS, categorized by median (> = median vs below)

^4^ β_G, beta coefficients for main effects of PRS

^5^ β_Interaction, beta coefficients for interaction effects between PRS and MVPA

### Calcium intake– genetic interaction on BMD

No statistically significant associations were found between calcium intake and BMD, but dietary calcium/phosphorous ratio was positively associated with total body BMD (*p* = 0.02). Also, no genome-wide or exome-wide significant genetic variants were identified for calcium intake and dietary calcium/phosphorous ratio. We also found no statistically significant interaction effects between PRS and calcium intake or dietary calcium/phosphorous ratio in association with total body BMD or lumbar spine BMD. However, in the secondary analysis with individual SNPs, we found significant interaction effects between calcium/phosphorous ratio and rs370055571 of Mitogen-Activated Protein Kinase Kinase Kinase Kinase 3 (*MAP 4 K3*) in relation to total body BMD (*p* = 0.02) and rs452369 of *SLC8A1* in relation to lumbar spine BMD (*p* = 0.036). In sensitivity analysis with secondary PRS, we found statistically significant interaction effects of lumbar spine BMD PRS with binary dietary calcium/phosphorous ratio on lumbar spine BMD (*p* = 0.037), indicating that high dietary calcium/phosphorous ratio was more beneficial in children with higher genetic susceptibility to low lumbar spine BMD as compared with children with lower genetic risk (Additional file 1).

All individual SNPs interaction results were in Additional file 1.

## Discussion

Our study addresses the research gap to investigate the effects of interaction between PRSs and calcium intake and physical activity on BMD in Hispanic children. We found higher MVPA to be more beneficial for children with higher genetic predisposition to low bone density in comparison to children less prone to low bone density. No statistically significant interaction effects on BMD were found between genetic risk and calcium intake or dietary calcium/phosphorous ratio.

Most studies that investigated interaction between physical activity and genetic variants used a candidate gene approach in primarily adult populations [6]. Studies have found that estrogen receptor-alpha (*ESRα*) polymorphisms may modulate the effect of exercise on BMD at weight bearing sites in children [29]. The vitamin D receptor (*VDR*) genotypes were also attributed to bone metabolic response variance following resistance training in young adult males [30]. One recent study in European children constructed a PRS based on GWAS-identified BMD loci in adults and found no evidence of effect of physical activity-PRS interactions on BMD, but did find beneficial effects on BMD of high impact physical activity on children overall with high genetic risk of low BMD [8]. We, on the contrary, found significant evidence of interaction between MVPA and PRSs in relation to total body BMD and lumbar spine BMD. Moreover, our results suggest that higher MVPA may benefit the BMD of children genetically susceptible to low BMD, although it may not provide meaningful benefit to children less genetically susceptible to low BMD. It is possible that these genes influence the bones’ responses to physical activity or physical activity changes the way these genes are expressed. Larger sample sizes may be needed to detect the interaction effects between continuous MVPA variables and PRS on BMD, compared to binary MVPA. In individual SNPs analysis, significant effect of interaction between MVPA and *SLC8A1*, *SEM1* loci and rs914153 were found to associate with BMD. Both *SLC8A1* and *SEM1* have been reported to associate with BMD or fractures in GWAS studies [17, 31]. Exercise training was found to reduce *SLC8A1* expression in animals susceptible to heart diseases [32] and *SLC8A1* was associated with hand grip strength and bone density [17, 33]. Exercise training was also found in mice to affect ubiquitin-proteasome systems [34], which has a role in skeletal muscle remodeling during exercise [35]. As skeletal muscle and muscle strength could influence bone strength [36], exercise might influence *SLC8A1* expression and interact with *SEM1* to affect ubiquitin-proteasome systems to further affect bone density. The SNP rs914153 is a novel locus that has not been reported to be associated with bone health or physical activity, so the mechanism is largely unknown. Given the scarcity of transcriptomic and epigenomic data from bone tissue [37], further research is needed to determine the functional impact of these genetic variants.

We did not find any statistically significant interaction between PRS and calcium intake or dietary calcium/phosphorous ratio to affect total body BMD and lumbar spine BMD. Calcium intake and dietary calcium/phosphorous ratio are mainly below the recommendation levels in our study and the genetic variants in PRS may exert more influence on BMD with higher calcium intake. Also, calcium intake was found in randomized controlled trials in pre-pubertal children to mainly increase BMD at appendicular skeletal sites but not lumbar spine potentially due to different calcification proportion in different sites [38, 39], which may partly explain the null interaction results on lumbar spine BMD. As PRSs only included several SNPs and our sample size is small, more genetic variants and larger sample size may be needed to detect the interaction effects. In individual variants analysis, we observed statistical interactions between dietary calcium/phosphorous ratio with *MAP 4 K3* and *SLC8A1* loci in relation to total body BMD and lumbar spine BMD respectively. As far as we know, no previous literature has reported the association between *MAP 4 K3* and bone health. However, the interaction is biologically plausible. *MAP 4 K3* encodes a kinase that is involved in the MAPK signaling pathway, which is critical to skeletal developments and maintenance and regulated by calcium [40]. *SLC8A1*, which encodes a sodium/calcium exchanger, was found to be associated with heel BMD and total body BMD [17]. Not all individual SNPs included in the PRS were found to interact with MVPA, calcium intake or calcium/phosphorous ratio probably due to involvements in different biological pathways or small effect sizes.

Our study has a few limitations. First, DXA-derived BMD is areal BMD (g/cm^2^) rather than volumetric BMD; although it is commonly used and considered as the gold standard to assess pediatric bone health, areal BMD tends to be lower for smaller bones compared to larger bones, even when the volumetric BMD is the same [24]. Also, even though total body excluding head is recommended, we used total body BMD as outcome due to SNPs associated with total body BMD may be more appropriate to assess variation from childhood to old age [17]. Second, there are some challenges to measure dietary intake in children accurately including parents’ ability to report young children’s food intake, children’s reliability to recall food intake, and adolescents’ concerns about body image, which results in greater within-between subject variances in nutrient intake in children compared to adults [41]. Although 2 days measurement of calcium intake were estimated to have good accuracy in adults [42], more days may be required to capture the usual intake of calcium. Moreover, we did not capture the specific type of physical activity in accelerometers, especially, weight-bearing physical activity, which is well-known to be beneficial to BMD. But MVPA is more likely to capture the weight-bearing physical activity compared to light or sedentary physical activity. Our accelerometer may also deviate from the current recommendations [43]. Also, the lumbar spine was not analyzed from a spine densitometry. Furthermore, although we account for main factors including age, sex, puberty stage, and BMI Z-scores, our results may not be comparable to all children studies on BMD due to the inconsistent covariates adjustment across studies and there could still be residual confounding [44]. Additionally, the sample size is small and may suffer from false positive in the results. Future studies are needed to replicate and validate our findings. Also, limited number of SNPs are included and the conclusions could be different if different SNPs are included in the PRS. Finally, as we used cross-sectional data in the analysis, the results may not reflect causal processes and future research in longitudinal studies is needed.

Our study also has many strengths. To our knowledge, this is the first study that investigated interactions between dietary calcium and physical activity with PRS affecting BMD in Hispanic children. First, we constructed the PRS based on literature and our association results from GWAS and exome sequencing data, which could account for joint effects of multiple genetic variants and improve power for detection of interactions [45]. Second, we created site-specific PRSs for interaction analysis due to genetic specificity to skeletal site [20], which may provide insights on how environments associate with bone density in people with skeletal site-specific genetic risk. Third, we had physical activity data measured by accelerometers, which is an objective, nonreactive approach to estimate physical activity compared to self-reported questionnaires [46].

In summary, MVPA is potentially more beneficial to bone health in Hispanic children who are at greater genetic risk for lower bone density. Future research on gene by environment interaction on bone health and functional studies to provide biological insights are needed.

## Supplementary Information

**Additional file 1:** **Supplemental Table 1.** Genetic Risk Score Compositions with only statistically significant BMD-associated SNPs ^a, b^. **Supplemental Table 2.** Baseline characteristics of children in the Viva La Familia Study by PRS. **Supplemental Table 3.** Interaction between PRS (with only statistically significant BMD-associated SNPs) and moderate to vigorous physical activity on TBBMD and LSBMD ^a, b, c, d^. **Supplemental Table 4.** Interaction between genetic markers and calcium intake, dietary calcium/phosphorous ratio on TBBMD and LSBMD ^a, b, c, d^.

## Data Availability

Data described in the manuscript, code book and analytic code will be made available upon request pending approval with corresponding author and all sequencing data described in the manuscript is publically available without restriction at https://www.ncbi.nlm.nih.gov/projects/gap/cgi-bin/study.cgi?study_id=phs000616.v2.p2.

## Abbreviations

BMD: Body mineral density
MVPA: Moderate to vigorous physical activity
SNP: Single nucleotide polymorphisms
DXA: Dual energy X-ray absorptiometry
PRS: Polygenic Risk Score
*MAP 4K3*: Mitogen-Activated Protein Kinase Kinase Kinase Kinase 3
*SLC8A1*: Solute Carrier Family 8 Member A1
*SEM1*: 26S Proteasome Complex Subunit
*VDR*: Vitamin D receptor
*IL6*: Interleukin-6
*ESR1*: Estrogen receptor α
VFS: The Viva La Familia Study
