# Supplementary material for: Genetic variants and physical activity interact to affect bone density in Hispanic children
Source: BMC Pediatr. 2021 Feb 15;21:79. doi: 10.1186/s12887-021-02537-y (PMC7883422; doi:10.1186/s12887-021-02537-y)
Supplement: Supplementary file 1 — Additional file 1: Supplemental Table 1. Genetic Risk Score Compositions with only statistically significant BMD-associated SNPs a, b. Supplemental Table 2. Baseline characteristics of children in the Viva La Familia Study by PRS. Supplemental Table 3. Interaction between PRS (with only statistically significant BMD-associated SNPs) and moderate to vigorous physical activity on TBBMD and LSBMD a, b, c, d. Supplemental Table 4. Interaction between genetic markers and calcium intake, dietary calcium/phosphorous ratio on TBBMD and LSBMD a, b, c, d. [file 12887_2021_2537_MOESM1_ESM.docx]

**Genetic variants and physical activity interact to affect bone density in Hispanic children**

Ruixue Hou^1^, PhD, Shelley A. Cole^2^, PhD, Mariaelisa Graff^3^, PhD, Yujie Wang^3^, PhD, Karin Haack^2^, PhD, Sandra Laston^4^, RN, PhD, Nitesh R. Mehta^5^, MS, Roman J. Shypailo^5^, Margaret L. Gourlay^6^, PhD, Anthony G. Comuzzie^7^, PhD, Kari E. North^3^, PhD, Nancy F. Butte^5^, PhD, Venkata Saroja Voruganti^1*^, PhD

1. Department of Nutrition and Nutrition Research Institute, University of North Carolina at Chapel Hill, Kannapolis, NC, USA (RH, VSV)

2. Texas Biomedical Research Institute, San Antonio, TX, USA (SAC, KH)

3. Department of Epidemiology, University of North Carolina at Chapel Hill, Chapel Hill, NC, USA (MS, YW, KEN)

4. South Texas Diabetes and Obesity Institute and Department of Human Genetics, University of Texas of the Rio Grande Valley, Brownsville, TX, USA (SL)

5. USDA/ARS Children’s Nutrition Research Center, Department of Pediatrics, Baylor College of Medicine, Houston, TX, USA (NRM, RJS, NFB)

6. Department of Family Medicine, University of North Carolina at Chapel Hill, Chapel Hill, NC, USA (RLG)

7. The Obesity Society, Silver Spring, MD, USA (AGC)

**Corresponding author:**

Venkata Saroja Voruganti

Address: Department of Nutrition and UNC Nutrition Research Institute, University of North Carolina at Chapel Hill, 500 Laureate Way, Kannapolis, NC 28081.

Telephone Number: 704-250-5009

Email Address: [saroja@unc.edu](mailto:saroja@unc.edu)

Supplemental Table 1. Genetic Risk Score Compositions with only statistically significant BMD-associated SNPs ^a, b^

| Marker | Gene | Risk Allele (Freq) | Chr | Position | Genetic Risk Score |
| --- | --- | --- | --- | --- | --- |
| rs452369 | *SLC8A1* | A(0.24) | 2 | 40157593 | Total body BMD PRS |
| rs529125 | *SUPT3H* | A(0.87) | 6 | 44829351 | Total body BMD PRS |
| rs2800718 | *LOC105377989* | A(0.21) | 6 | 127079180 | Total body BMD PRS |
| rs10953178 | *SEM1* | G(0.36) | 7 | 96501326 | Total body BMD PRS |
| rs950083 |  | C(0.74) | 10 | 122717980 | Total body BMD PRS |
| rs12273330 | *DGKZ* | A(0.03) | 11 | 46340356 | Total body BMD PRS |
| rs9846561 | *LEKR1* | A(0.96) | 3 | 157040993 | Lumbar spine BMD PRS |
| rs16934799 | *KCNMA1* | A(0.98) | 10 | 77448298 | Lumbar spine BMD PRS |
| rs2087058 | *LOC105369777* | G(0.54) | 12 | 54199621 | Lumbar spine BMD PRS |

^a^ Genetic risk score only includes SNPs that are statistically significant associated with BMD in Viva La Familia Study.

^b^ *DGKZ*, Diacylglycerol Kinase Zeta; *KCNMA1*, Potassium Calcium-Activated Channel Subfamily M Alpha 1; *LEKR1*, Leucine, Glutamate and Lysine Rick 1; *SEM1*, 26S Proteasome Complex Subunit; *SLC8A1*, Solute Carrier Family 8; *SUPT3H*, SPT3 Homolog, SAGA and STAGA Complex Component.

Supplemental Table 2. Baseline characteristics of children in the Viva La Familia Study by PRS

|  | **Overall** | **Male** | **Female** |  |  | **High TBBMD PRS** | **Low TBBMD PRS** | **High LSBMD PRS** | **Low LSBMD PRS** |
| --- | --- | --- | --- | --- | --- | --- | --- | --- | --- |
| Age, years | 11.0±4.1 | 11.2±3.9 | 10.7±4.2 |  |  | 11.3±3.8 | *10.5±4.2 | 11.1±3.9 | 10.8±4.0 |
| BMI, kg/m^2^ | 25.1±7.6 | 25.9±7.9 | *24.3±7.3 |  |  | 25.5±7.5 | 24.9±7.8 | 25.1±7.0 | 25.3±8.1 |
| Puberty Stage, Pre-pubertal % | 45.3 | 48.7 | *41.9 |  |  | 41.5 | *48.9 | 44.6 | 44.5 |
| Gender, Female% | 50.5 | NA | NA |  |  | 52.5 | 47.9 | 49.6 | 50.8 |
| **Dietary Intake** |  |  |  |  |  |  |  |  |  |
| Calcium intake, mg | 881.5±377.3 | 962.5±389.3 | *802.0±347.7 |  |  | 872.2±390.2 | 899.1±361.5 | 904.4±386.6 | 875.6±380.1 |
| Phosphorus intake, mg | 1180.3±407.8 | 1289.5±410.7 | *1073.0±375.5 |  |  | 1173.9±397.2 | 1201.0±420.5 | 1207.5±429.0 | 1175.2±391.1 |
| Dietary Calcium/Phosphorus ratio | 0.75±0.19 | 0.75±0.19 | 0.74±0.18 |  |  | 0.73±0.18 | *0.76±0.18 | 0.75±0.18 | 0.74±0.18 |
| **Physical Activity** |  |  |  |  |  |  |  |  |  |
| Moderate and Vigorous, mins/d | 82.3±51.8 | 90.2±52.9 | *74.5±49.5 |  |  | 80.2±52.0 | 87.7±25.9 | 87.9±53.6 | *79.2±50.9 |
| **Bone Mineral Density** |  |  |  |  |  |  |  |  |  |
| TBBMD, g/cm^2^ | 0.918±0.140 | 0.929±0.140 | *0.906±0.140 |  |  | 0.910±0.131 | 0.925±0.149 | 0.913±0.136 | 0.924±0.144 |
| LSBMD, g/cm^2^ | 0.805±0.184 | 0.785±0.176 | *0.825±0.191 |  |  | 0.796±0.177 | 0.810±0.189 | 0.784±0.176 | *0.824±0.188 |

^1^Values are means ± SDs or percentages.

*Indicated statistically significant differences between the groups (p<0.05)

Supplemental Table 3. Interaction between PRS (with only statistically significant BMD-associated SNPs) and moderate to vigorous physical activity on TBBMD and LSBMD ^a, b, c, d^

| Marker | β_G ^e^ | Pvalue_G | β_Interaction ^f^ | Pvalue_Interaction |
| --- | --- | --- | --- | --- |
| **TBBMD** |  |  |  |  |
| TBBMD_PRS, cat | -0.59 | 3.2×10^-6^ | 0.45 | 5.1×10^-4^ |
| **LSBMD** |  |  |  |  |
| TBBMD_PRS, cat | -0.55 | 2.3×10^-7^ | 0.45 | 4.6×10^-5^ |

^a^ Genetic risk score only includes SNPs that are statistically significant associated with BMD in Viva La Familia Study.

^b^ Only results with statistically significant interactions are shown (p value for interaction<0.05).

^c^ Adjusted for age, sex, BMI Z-score, and puberty stage.

^d^ LSBMD, lumbar spine bone mineral density; PRS, genetic risk score; TBBMD, total body bone mineral density; TBBMD_PRS, cat, TBBMD unweighted PRS, categorized by median (>=median vs below).

^e^ β_G, beta coefficients for main effects of PRS.

^f^ β_Interaction, beta coefficients for interaction effects between PRS and MVPA.

Supplemental Table 4. Interaction between genetic markers and calcium intake, dietary calcium/phosphorous ratio on TBBMD and LSBMD ^a, b, c, d^

| **Marker** | **β_G^5^** | **Pvalue_G** | **β_Interaction^6^** | **Pvalue_Interaction** |
| --- | --- | --- | --- | --- |
| **Dietary Calcium/Phosphorous Ratio, binary** | | |  |  |
| **Polygenic Risk Scores** |  |  |  |  |
| **LSBMD** |  |  |  |  |
| LSBMD_PRS, cat | -0.47 | 1.2×10^-5^ | 0.28 | 0.037 |
| **Individual SNPs** |  |  |  |  |
| **TBBMD** |  |  |  |  |
| rs370055571 | -0.97 | 2.1×10^-3^ | 1 | 0.02 |
| **LSBMD** |  |  |  |  |
| rs452369 | -0.09 | 0.42 | -0.28 | 0.036 |

^a^ Genetic risk score only includes SNPs that are statistically significant associated with BMD in Viva La Familia Study.

^b^ Only results with statistically significant interactions are shown (p value for interaction<0.05).

^c^ Adjusted for age, sex, BMI Z-score, and puberty stage.

^d^ LSBMD, lumbar spine bone mineral density; LSBMD_PRS, cat, LSBMD unweighted PRS, categorized by median (>=median vs below); PRS, genetic risk score; TBBMD, total body bone mineral density.

^e^ β_G, beta coefficients for main effects of PRS or individual SNPs.

^f^ β_Interaction, beta coefficients for interaction effects between PRS or individual SNPs and calcium intake or dietary calcium/phosphorous ratio.
